# The Mallet

**Published:** 1860-10

**Authors:** 


*The Mallet.*—We have been a little surprised to see this instrument recommended in the operation of filling teeth. Nothing can be more unnecessary and unsafe than its use in many of the delicate operations of the mouth. It must be awkward, unseemly, and calculated to alarm many patients, besides, being incapable of securing any real benefit over a masterly use of proper instruments. The epithet of "tooth carpenters" will be less inapplicable, if such singular implements become common in dental offices. B.

